# Degenerated uterine fibroid mimicking hydrometra: fallacy in CT

**DOI:** 10.2349/biij.2.4.e42

**Published:** 2006-10-01

**Authors:** CH Tok, SI Bux, SI Mohamed, BK Lim

**Affiliations:** 1 Department of Biomedical Imaging, Faculty of Medicine, University of Malaya, Kuala Lumpur, Malaysia; 2 Department of Obstetric and Gynaecology, Faculty of Medicine, University of Malaya, Kuala Lumpur, Malaysia

**Keywords:** CT, fibroids, hydrometra

## Abstract

Fibroids are the commonest uterine neoplasms, occurring in 20% - 30% of women of reproductive age. In women who have pelvic masses of unknown cause, unusual manifestations of fibroids such as necrosis or degeneration may simulate a carcinoma or hydrometra resulting in problems with image interpretation. We report a case of an unsuspected large degenerated uterine fibroid in a lady mistakenly diagnosed as hydrometra on computed tomography scanning.

## CASE REPORT

A 34-year-old nulliparous woman presented with sudden onset of severe abdominal pain and a six months history of progressive abdominal swelling. Her menstruation cycle was normal and regular. 5 years prior to admission she had undergone a right oophorectomy for a benign mucinous cystadenoma. On admission she was pale (Hb 9.4g/dl) with a large firm, tender and fixed pelvic mass corresponding to 36-week gravid uterus. Urine pregnancy test and serum tumour markers were negative. A clinical diagnosis of twisted ovarian cyst was made.

**Figure 1:**
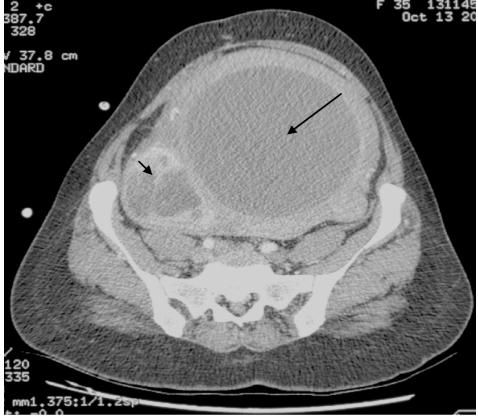
Contrast-enhanced axial CT abdomen demonstrating a large oval hypodense lesion [HU 30-70] (long arrow) within a large lobulated uterus with stretching of the myometrium. This is histopathologically proven as degenerated leiomyoma with large areas of necrosis and hyalinization, and inflammation is noted in both the fibroid and the myometrium, which simulates an endometrial fluid collection or hydrometra. A smaller separate heterogeneously enhancing lesion with whorl appearance (short arrow) is seen to the right side of the uterus and which is proven early degenerated leiomyoma.

Contrast enhanced spiral CT scan of the thorax, abdomen and pelvis revealed an enlarged lobulated uterus with a large (17.1 x 16.0 x 20 cm) fairly oval central hypodense lesion (30 – 70 HU) stretching the myometrium (Figure 1). In addition, there were several enhancing oval lesions noted in the lower uterine wall and a separate smaller inhomogeneously enhancing lesion (6.2 x 5.1 x 10 cm) in the right adnexa. The cervix was normal. There were no para-aortic, pelvic or inguinal lymphadenopathy and no ascites. The liver, spleen, pancreas and kidneys were normal. A diagnosis of a hydrometra with a right adnexal pathology was made.

**Figure 2:**
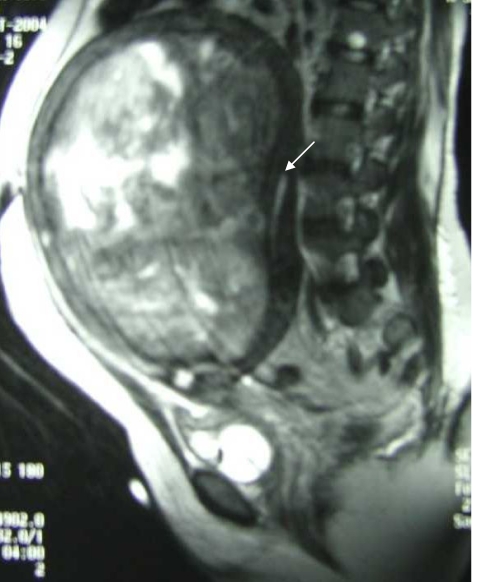
Sagittal T2-weighted section of MRI pelvis demonstrating large low signal intensity mass with multiple areas of high signal intensity within it arising from the anterior uterine wall. The normal endometrial stripe (white arrow) noted with normal thickness, differentiates the mass from gross hydrometra.

**Figure 3:**
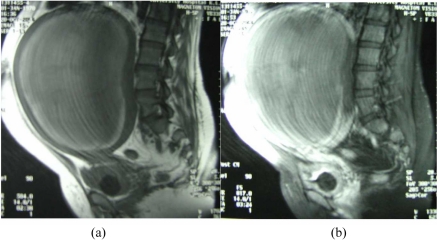
Pre-gadolinium-DTPA sagittal T1-weighted image of MRI pelvis (a) and post-gadolinium-DTPA image (b) demonstrating a large homogeneous low signal intensity lesion in the anterior uterine wall (arrow) in keeping with fibroid, which does not enhance following contrast administration. Motion artifacts are noted on post-gadolinium sequences showing in Figure 3(b).

Magnetic resonance (MR) imaging of the pelvis was performed to determine the cause of endocervical canal obstruction. It showed an enlarged uterine outline with 2 masses; a large left mass occupying almost the entire anterior wall of the uterus with compression and displacement of the endometrium posteriorly (Figure 2), and a smaller right mass. Both masses showed areas of high signal intensity on the T2WI, homogeneous low signal intensity on the T1WI and peripheral wall enhancement on the post-Gadolinium DTPA T1-weighted images (Figure 3). Features were consistent with that of degenerating fibroids.

**Figure 4:**
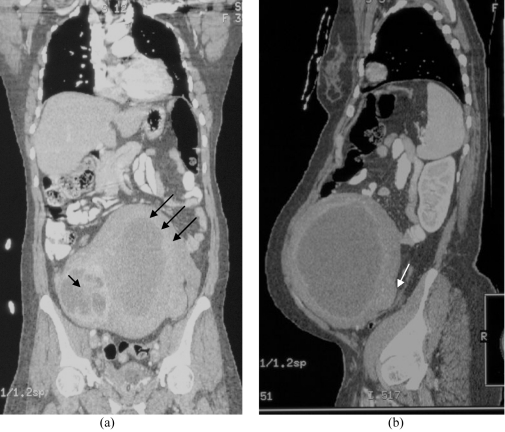
Retrospective reconstructed CT images in (a) coronal plane and (b) sagittal plane showing a large fairly oval hypodense lesion with surrounded ring enhancement (arrows) at the left side of the enlarged uterus. This is histopathologically proven as inflammation in the fibroid itself and the myometrium. There are a lot of blood vessels in the posterior uterine wall supplying to this lesion. Another smaller heterogenously enhancing lesion (short arrow) is seen on the right side of the uterus which appeared separate from the larger collection. (b) The endometrial lining cannot be identified on the sagittal plane. Another smaller homogeneously enhancing lesion (arrow) is seen at the posterior uterine wall, contributing to a nodular contour.

Retrospective 3D multiplanar reformation (MPR) of the CT dataset, confirmed the MRI findings. In addition numerous blood vessels were noted in the posterior uterine wall supplying the lesions (Figure 4).

Patient had total abdominal hysterectomy following failure of a trial of gonadotropin-releasing hormone (GnRH) analogues. Laparotomy confirmed multiple large uterine fibroids. Histopathological examination showed well-circumscribed fibroids with large areas of necrosis and hyalinisation with surrounding inflammation. The endocervical glands and the endometrium were normal. A diagnosis of multiple fibroids with a large degenerated component was made. Patient recovered well.

## DISCUSSION

Fibroids are classified as submucosal, intramural or subserosal based on their location. Intramural fibroids are within the substance of the myometrium and are the most common type but often asymptomatic. It has been estimated that 20%-50% of women with fibroids present with symptoms such as menorrhagia, dysmenorrhea, pressure, urinary frequency, pain, infertility, or a palpable abdominal / pelvic mass [1]. Pain occurs in approximately 30% of women with uterine fibroids and is usually the result of acute degeneration [1]. Degeneraton in fibroids, which occurs secondary to inadequate blood supply, may be hyaline (commonest), myxomatous, cystic, fatty, haemorrhagic or malignant in nature. The type of degenerative change seems to depend on the degree and rapidity of the onset of vascular insufficiency. GnRH analogues affect only undegenerated fibroids, and are ineffective when there is degeneration [2] as was noted in our patient

Even though fibroids are often found incidentally on CT, it is not the primary modality for diagnosing or evaluating fibroids. The common CT features include; uterine enlargement with a lobulated outer contour, focal myometrial thickening, deformed endometrial cavity, foci of punctate or amorphous coarse calcifications, and abnormal density within the soft tissue mass [3]. A fibroid may be hypodense, isodense or hyperdense relative to normal contrast-enhanced myometrium on CT scanning. Hyaline degeneration may be accompanied by varying degree of liquefaction thus resulting in a more cystic appearance [4] as was noted in our patient. A parasitized blood supply from an enlarged uterine artery may be responsible for the rare “hyperdense” fibroid [3]. Fast volume scanning in CT enables gapless data acquisition and overlapping images, thus increasing spatial resolution and reducing partial-volume averaging. 3D MPR enables display of a section of organs in a coronal or sagittal projection and thus depicting the anatomical structures more precisely hence reducing misinterpretation.

Other differential diagnoses of a hypodense lesion in the uterus on CT include leiomyosarcoma and hydrometra. Leiomyosarcoma occur in less than 1% of all cases and is impossible to distinguish it from a benign degenerating fibroid on imaging [5]. Hydrometra on CT appears as a symmetrically enlarged uterus, with a low-attenuation, non-enhancing central mass [6] while submocosal fibroids may asymmetrically distort or obliterate the uterine cavity [5] as was noted in our patient

In conclusion, fibroid degeneration with varying degree of liquefaction may simulate a hydrometra on CT. Recognizing the CT features on both hyaline degenerated fibroid and hydrometra are important. The application of 3D MPR, we believe can improve interpretation and diagnostic accuracy of CT.

**Table 1:** Differential characteristics of hydrometra and hyaline degenerated fibroid in the uterus on CT

**Hydrometra**	**Hyaline degenerated fibroid**
Symmetrically enlarged uterus [5]	Enlarged uterus with nodular contour, submucosal fibroids may asymmetrically distort or obliterate the uterine cavity
Homogeneous enhanced myometrium	Diminished contrast enhanced of the myometrium and showing ring enhancement
Non-enhancing central mass [5]	May be accompanied by varying degree of liquefaction [3]; thus more cystic appearance
Usually secondary to cervical canal obstruction	Can localize elsewhere
